# Partial attenuated PTH suppression of calcimimetics may be due to the resulting hypocalcemia or a significant decrease in serum calcium levels within the normal reference range without adequate calcium supplementation

**DOI:** 10.3389/fmed.2026.1907882

**Published:** 2026-09-30

**Authors:** De-ru Kong, De-ya Kong, Li-yuan Mu, Xiu-quan Qu, Xi-rui Li, Ling-quan Kong

**Affiliations:** 1 Department of Breast and Thyroid Surgery, The First Affiliated Hospital of Chongqing Medical University, Chongqing, China; 2 Xiangya School of Medicine, Central South University, Changsha, China

**Keywords:** apparent resistance, attenuated PTH suppression, calcimimetic agents, calcium-sensing receptor, hungry bone syndrome, hypocalcemia, parathyroid hormone, secondary hyperparathyroidism

## Abstract

Certain dialysis patients with secondary hyperparathyroidism (SHPT) show partial insensitivity to calcimimetics. These agents are allosteric activators of the calcium-sensing receptor (CaSR) and enhance their sensitivity to extracellular calcium, thereby inhibiting parathyroid hormone (PTH) secretion and eliciting a decrease in serum calcium levels. We hypothesize that the partial attenuated PTH suppression of calcimimetics may be due to the resulting hypocalcemia or a significant decrease in serum calcium levels within the normal range without adequate calcium supplementation. Effective suppression of PTH by calcimimetics inevitably lowers serum calcium levels, which may be further aggravated by ongoing bone mineralization following PTH suppression. This hypocalcemic state or decreased extracellular ionized calcium, if not adequately corrected, acts as a strong trigger for excessive PTH secretion through reducing CaSR activation and stimulating parathyroid chief cells and causing hypertrophy and hyperplasia of parathyroid glands, and then establishing a counter-regulatory feedback circuit that diminishes the therapeutic effect of calcimimetics. High incidence of hypocalcemia, observed in clinical trials of calcimimetics, also bolsters the validity of this hypothesis. Such adverse outcomes may be partially alleviated by timely and adequate calcium and vitamin D supplementation.

## Introduction

Chronic kidney disease-mineral and bone disorder (CKD-MBD) is a systemic mineral and bone metabolism disorder caused by CKD. The disease is often accompanied by serious complications such as fragility fractures and vascular and valvular calcification (1). Secondary hyperparathyroidism (SHPT) is one of the most common types of CKD-MBD, with an incidence of 11.5%–54%. It is characterized by excessive parathyroid hormone (PTH) secretion, resulting in high morbidity and mortality (1–3). Calcimimetics, such as cinacalcet, are widely used in the management of moderate to severe SHPT in patients undergoing dialysis (4). As allosteric activators of calcium-sensing receptor (CaSR), calcimimetics can enhance CaSR's sensitivity to extracellular calcium, thus inhibiting the release of PTH (5) and eliciting a decrease in serum calcium concentrations (6, 7). Researches suggested cinacalcet reduced the rate of parathyroidectomy by more than half (8).

However, some patients do not respond adequately and are ultimately referred to a parathyroid surgeon to discuss parathyroidectomy (9). Data from the Evaluation of Cinacalcet Hydrochloride Therapy to Lower Cardiovascular Event (EVOLVE) trial in secondary hyperparathyroidism patients receiving dialysis demonstrated that 58.3% of 1,938 patients randomized to cinacalcet experienced at least one episode of hypocalcemia versus 14.9% of 1,923 patients randomized to placebo (10). Part of the hypocalcemic action of cinacalcet in certain patients with high bone turnover exhibits pathophysiological similarities to hungry bone syndrome (HBS)–a disorder commonly noted following parathyroidectomy in individuals with severe SHPT, where rapid bone remineralization leads to profound and prolonged hypocalcemia (11–15). Prolonged hypocalcemia or significant decrease in serum calcium levels that is not corrected adequately and timely may directly cause reduced CaSR activation and stimulate parathyroid chief cells, leading to parathyroid hypertrophy and hyperplasia, and then increased secretion of PTH (16, 17). This may antagonize the efficacy of calcimimetics, thereby causing attenuated PTH suppression of calcimimetics (Figure 1).

**Figure 1:**
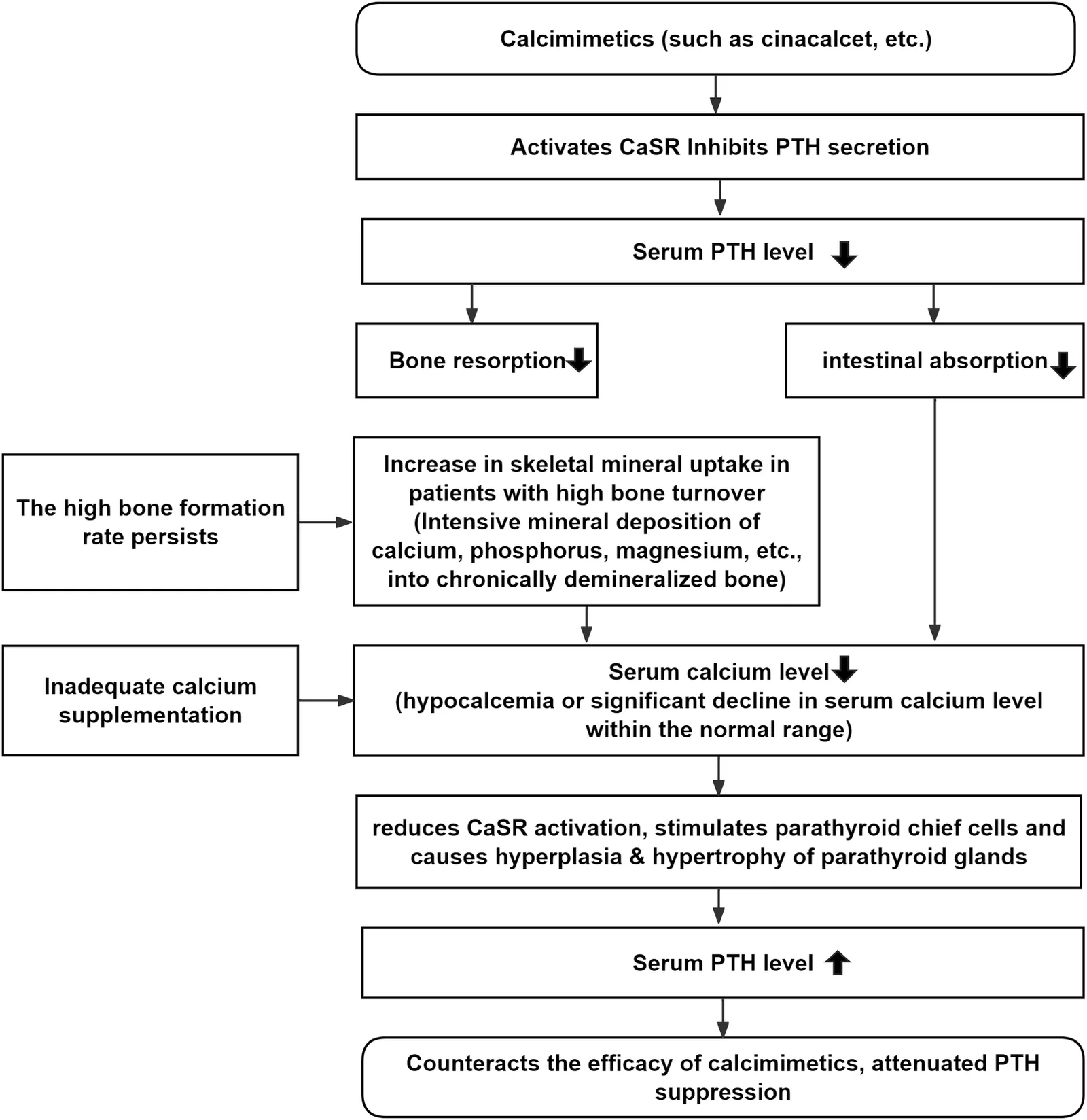
Proposed mechanism model of attenuated PTH suppression of calcimimetic in SHPT patients receiving dialysis. Calcimimetics activate CaSR to suppress PTH, reducing serum calcium mainly via decreased bone resorption, and intestinal absorption. The model hypothesizes that inadequate calcium supplementation prolongs hypocalcemia, which in turn leads to paradoxical PTH over-secretion through reducing CaSR activation and stimulating parathyroid chief cells and causing hypertrophy and hyperplasia of parathyroid glands (dashed loop), counteracting calcimimetic efficacy. Abbreviations: CaSR, calcium-sensing receptor; PTH, parathyroid hormone; SHPT, secondary hyperparathyroidism.

Notably further parathyroid hyperplasia and hypersecretion may lead to increased dosage of calcimimetics, resulting in more side effects, or necessitate discontinuation due to counter-regulatory PTH rebound, which are clinically preventable. Early identification of these secondary factors can effectively prevent hypocalcemia or significant decrease in serum calcium levels, subsequently reducing the risk of severe hypocalcemia-related complications and the development of calcimimetics-refractory SHPT. However, despite its clinical significance, this issue has not received sufficient attention in clinical practice.

## The pharmacological effect of calcimimetics inducing a reduction in serum PTH and calcium concentrations

Calcimimetics function as allosteric activators of CaSR. As such, they can increase the sensitivity of the CaSR to extracellular calcium ions and inhibit PTH secretion (5, 18). When calcimimetics effectively reduce the level of PTH, it will inevitably reduce the level of serum calcium through a variety of mechanisms including inhibiting bone absorption (16), and reducing intestinal calcium absorption (19). It was reported that calcimimetics potently reduced PTH levels and frequently resulted in hypocalcemia (8). In the EVOLVE trial, at least one episode of hypocalcemia occurred within 16 weeks after the initial dose among 58.3% of 1,938 individuals randomized to cinacalcet group compared with 14.9% of 1,923 individuals randomized to placebo group. Hypocalcemia was severe in 18.4% of the cinacalcet group versus 4.4% in the placebo group (10).

## Hypocalcemia or significant decrease in serum calcium level due to a possible increase in skeletal mineral uptake after administration of calcimimetics in some patients with high bone turnover

The decreases in serum calcium levels induced by calcimimetics may also partially result from the abrupt cessation of PTH-driven bone resorption while bone formation continues, causing a large amount of minerals (mainly calcium, phosphorus, and magnesium) to be deposited in chronically demineralized bone, and exacerbating the hypocalcemia (10, 16). Concomitant hypophosphatemia, hypomagnesemia, and hyperkalemia may also occur (11).

It was reported that a 55-year-old male patient with SHPT developed severe, prolonged symptomatic hypocalcemia 2 weeks after initiating cinacalcet therapy (13). Serum calcium reverted to the normal reference range after 4 weeks of discontinuing the drug while undergoing high doses of elemental calcium and active vitamin D therapy (13). It was also reported that a cinacalcet-treated SHPT patient on dialysis developed hypocalcemia and such pronounced hypophosphatemia and severe diffuse bone pain, and then it was recommended that the laboratory monitoring of cinacalcet-treated patients should include serum phosphate levels and not only intact PTH and calcium, as currently suggested (14). Although cinacalcet-induced hypocalcemia typically has a more gradual onset (10), it still presents substantial clinical risks. Transient hypocalcemia has been linked to an increased risk of cardiovascular mortality regardless of cinacalcet administration (20).

## Hypocalcemia or decrease in serum calcium level further stimulates parathyroid hyperplasia and counteracts the therapeutic effect of calcimimetics

A significant decrease in serum calcium concentrations or hypocalcemia caused by calcimimetics may further stimulate parathyroid, resulting in parathyroid hyperplasia, hypertrophy and hyperfunction (16, 17). It was reported that young female rats were fed with normal (1.18%) or low (0.05%) calcium diet for 3, 7, 15 or 30 days. The morphology of the parathyroid glands was studied together with serum calcium, parathyroid hormone (PTH), calcitonin and bone mineral density (BMD). As compared to the animals fed with the normal calcium diet, BMD of whole body of the rats fed with the low calcium diet was significantly decreased, whereas the serum PTH level was increased. The parathyroid glands in the rats fed with the low calcium diet were markedly enlarged. Enlargement of the cytoplasm and rather frequent mitoses of the parathyroid chief cells were observed in the rats fed with the low calcium diet for 15 and 30 days. These findings suggest that the rapid bone loss in young rats induced by the low calcium diet is essentially due to stimulated activity of the parathyroid gland. The stimulated gland may be a result of hypertrophy at the early stage and a combination of hypertrophy and hyperplasia at the later stage of calcium deficiency (17).

The parathyroid glands respond quickly to fluctuations in serum calcium levels. Research shows that even a slight decrease of ionized calcium levels can cause significant change in PTH levels (21). Shrestha et al. also presented a mathematical model of the Calcium-PTH axis and described the supports the rapid kinetics of PTH secretion in response to serum ionized calcium changes (22). Therefore, the hypocalcemia or a substantial decline in serum calcium level within the normal reference range induced by effective doses of calcimimetics will continue to stimulate the parathyroid glands by reducing CaSR activation, prompting them to secrete more PTH. This may lead to a rebound in the level of PTH or the inability to achieve further inhibition, which is manifested as a partial resistance to the drug.

## Avoidance and correction of clinically significant hypocalcemia or significant decline in serum calcium levels even within the normal reference range may help to prevent reduced CaSR activation

The parathyroid glands are extremely sensitive to changes in serum calcium levels, and even if the small changes in ionic calcium levels, may cause significant changes in the level of PTH (21). Therefore, avoidance and timely correction of clinically significant decrease in serum calcium levels (even within the normal reference range) may prevent reduced CaSR activation, reduce the stimulation of the parathyroid glands, enhance the therapeutic effect, and reduce the risk of apparent resistance.

This implies that avoidance and correction of clinically significant hypocalcemia while minimizing excessive calcium loading is the key to successful therapy. It was reported that high dialysate calcium may improve the therapeutic effect of calcimimetics (23).

All calcimimetics act as positive allosteric modulators of CaSR, and their PTH-lowering effect is fundamentally dependent on adequate extracellular calcium availability. Therefore, the core premise of our hypothesis—that insufficient calcium supplementation may limit the degree of PTH suppression—is mechanistically applicable to evocalcet and etelcalcetide as well. While our hypothesis is biologically plausible for all three agents, direct evidence is currently available mainly for cinacalcet. Future studies specifically evaluating evocalcet and etelcalcetide with predefined calcium supplementation protocols are warranted to confirm the generalizability of our findings.

Additionally, factors such as advanced parathyroid hyperplasia, reduced CaSR expression, medication adherence, and genetic variability may also contribute to treatment failure of calcimimetics. Other potential mechanisms of calcimimetic resistance includes: (1) Advanced parathyroid hyperplasia–which may reduce the functional reserve of parathyroid glands and limit responsiveness to calcimimetics; (2) Reduced CaSR expression – as downregulation of the target receptor could attenuate drug efficacy regardless of calcium availability; (3) Medication adherence – as suboptimal compliance is a common and often overlooked cause of apparent treatment failure in clinical practice; (4) Genetic variability – including polymorphisms in the CaSR gene or other components of the calcium-sensing pathway that may influence individual drug responses.

## Conclusion

On the basis of observations from the application of calcimimetics in certain dialysis patients with SHPT, we hypothesize that partial attenuated PTH suppression of calcimimetics may be due to treatment-induced hypocalcemia or substantial decline in serum calcium levels within the normal reference range in the absence of adequate calcium supplementation. The core of this hypothesis lies in the fact that, without avoidance and timely correction of clinically significant hypocalcemia while minimizing excessive calcium loading, the therapeutic efficacy of calcimimetics is inherently constrained by their key biochemical effects-lowering serum calcium concentration, which manifests as either overt hypocalcemia or clinically significant reductions in serum calcium levels within the normal reference range. To validate this hypothesis still requires further scientific research. The following approaches may be helpful: 1) Prospective clinical studies: Conduct studies in dialysis patients for prospective temporal analyses showing that calcium reduction precedes subsequent PTH rebound or reduced net PTH suppression, and initiate calcimimetics with different doses of calcium supplementation under close monitoring of serum levels of calcium and PTH. The decline in serum ionized calcium of ≥ 0.025 mmol/L from baseline, or a decline that reaches serum calcium < 2.1 mmol/L (< 8.5 mg/dL) within the first weeks may be taken as an exploratory threshold for prospective testing to show reduced net PTH suppression or even PTH rebound, compared to those with more stable calcium levels. 2) Analysis of existing trial data: Re-analysis of the data from large trials like EVOLVE. Correlate the magnitude and time-course of hypocalcemia episodes with the subsequent PTH levels and the need for dose escalation. The hypothesis would be supported if patients with severe or prolonged hypocalcemia were more likely to be classified as “inadequate responders”. 3) Mechanistic studies: Measure bone turnover markers (e.g., bone-specific alkaline phosphatase, C-terminal telopeptide of type I collagen) in patients receiving calcimimetics. The hypothesis predicts that a pattern consistent with suppressed bone resorption but persistent formation (an “uncoupling” akin to early HBS) would correlate with both greater hypocalcemia and poorer PTH suppression.

If this hypothesis is verified, the calcimimetic therapeutic strategy for SHPT will require a fundamental change–from a simple incremental calcimimetic dose to an active avoidance and timely correction of clinically significant hypocalcemia while minimizing excessive calcium loading.

The hypothesis provides a theoretical basis for initiation of combined treatment - that is, the combination of calcium/vitamin D to inhibit the hypocalcemia feedback loop while using calcimimetics, thus promoting better and lasting PTH inhibition. Mislabeling this physiology as “apparent resistance” in calcimimetics-treated patients due to inadequate calcium intake may create a vicious cycle. It may cause to increase the drug dose and worsen the condition or prompt unnecessary surgery. Such adverse outcomes may be effectively alleviated by timely and adequate calcium and vitamin D supplementation. It should also be noted that calcium supplementation in dialysis patients requires careful evaluation given the potential risks of vascular calcification and positive calcium balance.

## Limitations of the hypothesis

It should be noted that this theoretical hypothesis currently remains preliminary and is largely based on indirect observations, *post-hoc* interpretation of existing studies, and a small number of clinical reports, which may not be broadly generalizable; Therefore, our hypothesis should be viewed as exploratory and hypothesis-generating, and further prospective studies with predefined endpoints are warranted to test its validity.

## Data Availability

Publicly available datasets were analyzed in this study. This data can be found here: The analyzed datasets are derived from publicly available published clinical trials and peer-reviewed literatures cited in this manuscript. Readers can access the original data via the official websites of corresponding published articles. No unified public data repository or dedicated accession number is available.

## References

[1] He X Narushima K Kojima M Nagai C Li K. Pharmacokinetics, pharmacodynamics, and safety of evocalcet (KHK7580), a novel calcimimetic agent: an open-label, single- and multiple-dose, phase I trial in healthy Chinese subjects. Drug Des Devel Ther. 2024;18:567–81. 10.2147/DDDT.S437903 38436038 PMC10906727

[2] Palmer SC. Serum levels of phosphorus, parathyroid hormone, and calcium and risks of death and cardiovascular disease in individuals with chronic kidney disease: a systematic review and meta-analysis. JAMA. 2011;305(11):1119. 10.1001/jama.2011.308 21406649

[3] Chai Y Yuan N Yin J Shen B Sun L Zhang L. Machine learning-based predictive model for hungry bone syndrome following parathyroidectomy in secondary hyperparathyroidism. Front Endocrinol. (2025) 16:1635451. 10.3389/fendo.2025.1635451 PMC12446021 40979702

[4] Block GA Bushinsky DA Cheng S Cunningham J Dehmel B Drueke TB. Effect of etelcalcetide vs cinacalcet on serum parathyroid hormone in patients receiving hemodialysis with secondary hyperparathyroidism: a randomized clinical trial. JAMA. (2017) 317(2):156. 10.1001/jama.2016.19468 28097356

[5] Goodman WG Ward DT Martin KJ Drayer D Moore C Xu J. Activation of the calcium receptor by calcimimetic agents is preserved despite modest attenuating effects of hyperphosphatemia. J Am Soc Nephrol. (2022) 33(1):201–12. 10.1681/ASN.2021060825 34732508 PMC8763177

[6] Bover J Ureña P Ruiz-García C Dasilva I Lescano P Del Carpio J. Clinical and practical use of calcimimetics in dialysis patients with secondary hyperparathyroidism. Clin J Am Soc Nephrol. (2016) 11(1):161–74. 10.2215/CJN.01760215 26224878 PMC4702220

[7] Palmer SC Mavridis D Johnson DW Tonelli M Ruospo M Strippoli GFM. Comparative effectiveness of calcimimetic agents for secondary hyperparathyroidism in adults: a systematic review and network meta-analysis. Am J Kidney Dis. (2020) 76(3):321–30. 10.1053/j.ajkd.2020.02.439 32475604

[8] The EVOLVE Trial Investigators. Effect of cinacalcet on cardiovascular disease in patients undergoing dialysis. N Engl J Med. 2012;367(26):2482–94. 10.1056/NEJMoa1205624 23121374

[9] Steinl GK Kuo JH. Surgical management of secondary hyperparathyroidism. Kidney Int Rep. 2021;6(2):254–64. 10.1016/j.ekir.2020.11.023 33615051 PMC7879113

[10] Floege J Tsirtsonis K Iles J Drueke TB Chertow GM Parfrey P. Incidence, predictors and therapeutic consequences of hypocalcemia in patients treated with cinacalcet in the EVOLVE trial. Kidney Int. 2018;93(6):1475–82. 10.1016/j.kint.2017.12.014 29525393

[11] Lau WL Obi Y Kalantar-Zadeh K. Parathyroidectomy in the management of secondary hyperparathyroidism. Clin J Am Soc Nephrol. 2018;13(6):952–61. 10.2215/CJN.10390917 29523679 PMC5989682

[12] Silarat P Saeseow S Pathumarak A Srisuwarn P Suvikapakornkul R Disthabanchong S. Improved clinical outcomes associated with hungry bone syndrome following parathyroidectomy in dialysis patients. Endocr Pract. 2024;30(11):1079–88. 10.1016/j.eprac.2024.08.004 39214462

[13] Koubar SH Qannus AA Medawar W Abu-Alfa AK. Hungry bone syndrome two weeks after starting cinacalcet: a call for caution. CEN Case Rep. 2018;7(1):21–3. 10.1007/s13730-017-0284-z 29124559 PMC5886916

[14] Nowack R Wachtler P. Hypophosphatemia and hungry bone syndrome in a dialysis patient with secondary hyperparathyroidism treated with cinacalcet–proposal for an improved monitoring.Clin Lab.2006;52(11-12):583–7. 17175888

[15] Lazar ES Stankus N. Cinacalcet-induced hungry bone syndrome.Semin Dial. 2007;20(1):83–5. 10.1111/j.1525-139X.2007.00248.x 17244128

[16] Qu X-q Mu L-y Liang X-y Jiang Z-y Feng J-h She R-l. Partial primary osteoporosis maybe due to parathyroid hyperfunction caused by calcium intake insufficiency and/or vitamin D insufficiency/deficiency. Med Hypotheses. (2024) 182:111236. 10.1016/j.mehy.2023.111236

[17] Chen H Hayakawa D Emura S Ozawa Y Taguchi H Yano R. Effect of low calcium diet on the ultrastructure of the rat parathyroid gland. Okajimas Folia Anat Jpn. (2001) 78(5):153–9. 10.2535/ofaj1936.78.5_153 11915356

[18] Shoji T Nakatani S Kabata D Mori K Shintani A Yoshida H. Comparative effects of etelcalcetide and maxacalcitol on serum calcification propensity in secondary hyperparathyroidism: a randomized clinical trial. Clin J Am Soc Nephrol. (2021) 16(4):599–612. 10.2215/CJN.16601020 33685864 PMC8092049

[19] Lee JJ Liu X O'Neill D, Beggs MR, Weissgerber P, Flockerzi V Activation of the calcium sensing receptor attenuates TRPV6-dependent intestinal calcium absorption. JCI Insight. (2019) 5(11):e128013. 10.1172/jci.insight.128013 31013259 PMC6629117

[20] Goto S Hamano T Fujii H Taniguchi M Abe M Nitta K. Hypocalcemia and cardiovascular mortality in cinacalcet users. Nephrol Dial Transplant. (2024) 39(4):637–47. 10.1093/ndt/gfad213 37777840

[21] Blum JW Fischer JA Schwoerer D Hunziker W Binswanger U. Acute parathyroid hormone response: sensitivity, relationship to hypocalcemia, and rapidity. Endocrinology. 1974;95(3):753–9. 10.1210/endo-95-3-753 4850418

[22] Shrestha RP Hollot CV Chipkin SR Schmitt CP Chait Y. A mathematical model of parathyroid hormone response to acute changes in plasma ionized calcium concentration in humans. Math Biosci. (2010) 226(1):46–57. 10.1016/j.mbs.2010.04.001 20406649

[23] Touam M Menoyo V Attaf D Thebaud HE Drüeke TB. High dialysate calcium may improve the efficacy of calcimimetic treatment in hemodialysis patients with severe secondary hyperparathyroidism. Kidney Int. 2005;67(5):2065. 10.1111/j.1523-1755.2005.310_1.x 15840060

